# Case Report: Gut and spleen anomalies associated with DYRK1A syndrome

**DOI:** 10.3389/fped.2022.936732

**Published:** 2023-01-18

**Authors:** I. Infantino, F. Tocchioni, M. Ghionzoli, R. Coletta, F. Morini, A. Morabito

**Affiliations:** ^1^ Department of Neuroscience, Psychology, Drug Research and Child Health (NEUROFARBA), University of Florence, Florence, Italy; ^2^ Department of Pediatric and Neonatal Surgery, Meyer Children's Hospital IRCSS, Florence, Italy; ^3^ School of Health and Society, University of Salford, Salford, United Kingdom

**Keywords:** spleen abnormalities, gut abnormalities, DYRK1A, intestinal obstruction, splenectomy

## Abstract

DYRK1A syndrome has been extensively studied primarily with regard to neurologic and other phenotypic features such as skeleton and craniofacial alterations. In the present paper, we aim to highlight unusual anomalies associated with a DYRK1A mutation: a 17-year-old female patient with language and cognitive delay, microcephaly, and an autistic disorder, who was operated upon for spleen torsion with anomalous gut fixation.

## Background

DYRK1A is a gene located on the critical region of chromosome 21q22 (1). It codifies for a dual-specificity tyrosine kinase, enrolled in the phosphorylation of serine and threonine residues (2, 3) and tyrosine residues in their own loop (3). This protein is expressed in numerous systems and its role is pivotal, especially for neuronal differentiation, neurogenesis, and neurodegeneration (2) through MAP kinase activation. Because of these reasons, the main features of patients with DYRK1A mutations are intellectual disability, language delay, microcephaly, difficulties in feeding and thriving, developmental retardation, and seizures (4) as summarized in Table 1. Other major clinical features comprise autism spectrum disorder, anxiety, and sleeping disorders. Other frequent clinical features are related to craniofacial district and gastrointestinal tract alteration (3). These patients present with typical facial features: “moody” expression, deep set eyes, bitemporal narrowing, narrow nasal root, down-slanted palpebral fissures, dysplastic ears, and micrognathia (5). Associated skeletal malformations are described, such as pectus excavatum, scoliosis, syndactyly, and feet anomalies (6). Optic development is also impaired, and patients often present with strabismus, astigmatism, enophthalmia (7), and optic nerve atrophy (8). Dental anomalies and cardiac defects are described as well (6, 9). Overall, patients affected with DYRK1A mutations experience feeding difficulties, which are responsible for imbalances in weight gain after birth or for short stature (10). Interestingly enough, in the present paper, we report the case of a teenager who was operated upon for a spleen torsion with anomalous gut fixation and who was affected by DYRK1A syndrome.

**Table 1:** Data from van Bon BWM, Coe BP, de Vries BBA, et al. In: Adam MP, Ardinger HH, Pagon RA, et al. *DYRK1A syndrome*. Published online March 17, 2015, updated 2021, Table 2.

Feature	Frequency of person w/feature
DD/ID	100%
Hypertonia	12/33
Gait disturbances	24/45
Speech impairment	100%
Feeding problems	93%
Epilepsy	65%
ASD	46%
Anxiety	27%
Hyperactivity	10/35
Sleep disturbance	6/15
Microcephaly	95%
Weight < 2 SD	49%
Short stature	44%
Eye abnormalities	79%
Characteristic facial features	90%
Cardiac defects	9/48
Gastrointestinal problems	30%
Urogenital anomalies	40%
Musculoskeletal features	10%
Dental anomalies	6/36

DD, developmental disorder; ID, intellectual disability; ASD, autism spectrum disorder.

## Case report

A 17-year-old female patient affected by DYRK1A syndrome (autosomal-dominant DYRK1A *de novo* mutation, variant c.95I+5G>A), with language and cognitive delay, microcephaly, and an autistic disorder, presented to us through the emergency department for abdominal distension, retching, and bilious leakage from the gastrostomy tube.

Her past surgical history indicated patent ductus arteriosus ligation, percutaneous endoscopic gastrostomy placement plus Nissen fundoplication performed at age 3 for severe gastroesophageal reflux disease (GERD) subsequently complicated with diaphragmatic rupture requiring a redo surgery. She suffered from recurrent subocclusive episodes, which were managed with a conservative approach, and several bouts of pneumonia requiring hospitalization.

**Figure 1:**
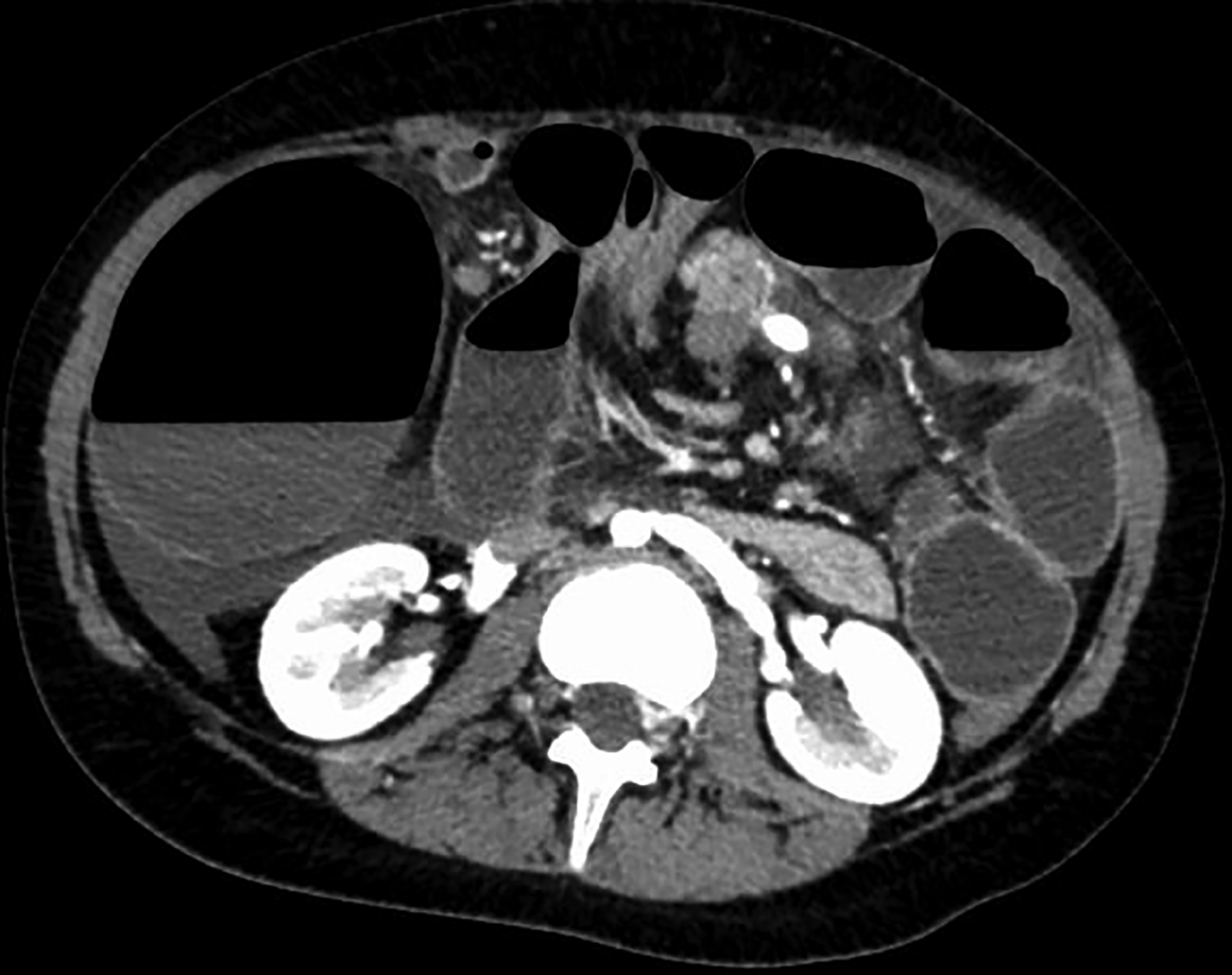
Transverse section of patient's CT scan upon admittance, showing whirpool sign of mesenteric vessels and dilated right colon with air-fluid levels.

**Figure 2:**
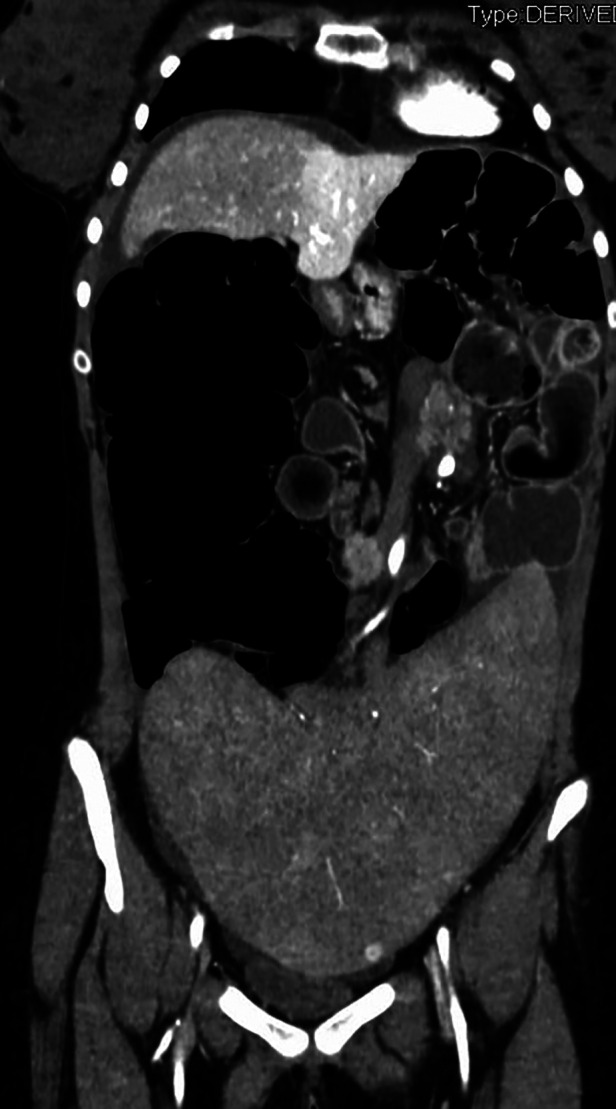
Coronal section of patient's CT scan upon admittance, showing enlarged spleen located in the pelvis with torsion of splenic vessels and distended right colon.

On admission, the plain film demonstrated signs of intestinal occlusion, and blood samples showed thrombocytopenia and hyperamylasemia. An abdominal CT scan revealed an enlarged twisted pelvic spleen associated with a spiral mesenteric root and a verticalized pancreatic body and tail. The ascending colon was dilated, while the hepatic flexure and transverse colon were stretched, as shown in Figures 1, 2.

The patient underwent an urgent laparotomy *via* median incision upon clinical and radiological evaluation. An enlarged pelvic spleen was found twisted without any signs of ischemia, dragging in its torsion a part of the pancreas. Considering the patient's age and an enlarged spleen bearing a high risk of ab extrinseco intestinal obstruction and recurrent torsion, a splenectomy was performed. Further abdominal assessment led to right colonic volvulus detection due to an absent right colonic fixation. Following its detorsion, the cecum appeared extremely dilated (diameter > 15 cm). Lastly, an extremely long sigmoid colon was found. No other intestinal fixation or position anomalies were found. Taking into consideration all the mentioned findings, an ascending colectomy with ileo-colic latero-lateral anastomosis, together with a sigmoidectomy with a colorectal latero-lateral anastomosis, was performed. The patient’s postoperative course was uneventful: an antibiotic prophylaxis, combined with a vaccine plan, was devised as in postsplenectomy care. After 10 days, the patient reached the full feed stage and had regular bowel movement, following which she was discharged.

## Discussion

In this short study, we aim to highlight unusual abdominal findings that have never been described in a patient affected by DYRK1A syndrome with a *de novo* genetic mutation, showing common features as in facial features and brain MRI findings (1).

Our bibliographic search tools included PubMed, Google Scholar, and Medline. DYRK1A AND acute abdomen, DYRK1A AND spleen, AND wandering spleen, AND bowel obstruction, AND obstructive episodes, AND abdominal pain were used as medical subject heading (MeSH) terms.

With regard to gastrointestinal features, significant gastro-esophageal reflux and severe constipation have been described in DYRK1A syndrome patients, requiring some of these patients to undergo the gastrostomy placement procedure, which is possibly associated with an antireflux procedure as in our case.

However, no cases of colic and splenic alterations have been reported previously.

Wandering spleen is a condition of congenital or acquired laxity of peritoneal ligaments (11), causing an abnormal spleen position in the abdomen and potential volvulus of the elongated vessels (12) associated with a torsion of the pancreatic tail (13). This is usually asymptomatic and often is an incidental imaging finding (14). In our patient, splenomegaly with associated thrombocytopenia and an altered position (in the lower abdomen) have been recorded for a period of 6 years.

In the course of the Nissen procedure and diaphragmatic rupture repair, no splenic ligament was resected, except for only two short gastric vessels, and no spleen abnormalities in position, rotation, and mobility were described at the time, ruling out the possibility of a postsurgical fixation defect.

A CT scan should be considered in patients with DYRK1A syndrome and presenting with recurrent subocclusive symptoms to better detail the abdominal anatomy.

Molecular studies on animal models have demonstrated the expression of DYRK1A in multiple organs and tissues, both in the embryo and postnatally, with the highest rates reported in the central nervous system (CNS) and heart and the lowest rates in the kidneys, liver, spleen (15) as well as the epithelial layers of the gut, stomach, and skeletal muscle (16). New theories about the dose dependence of DYRK1A in the pathogenesis of various clinical conditions (17) are being developed. It would be an interesting proposition to quantitatively investigate the residual expression of DYRK1A in the causative mutations of DYRK1A syndrome.

## Conclusions

Our aim was to highlight the peculiar abdominal anatomy associated with the DYRK1A syndrome phenotype to improve the management of such syndrome-affected patients in an attempt to avoid urgent surgery.

## Data Availability

The datasets for this article are not publicly available due to concerns regarding participant/patient anonymity. Requests to access the datasets should be directed to the corresponding author.

## References

[1] Luco SM Pohl D Sell E Wagner JD Dyment DA Daoud H. Case report of novel DYRK1A mutations in 2 individuals with syndromic intellectual disability and a review of the literature. BMC Med Genet. (2016) 17:15. 10.1186/s12881-016-0276-4 26922654 PMC4769499

[2] Meissner LE Macnamara EF D'Souza P Yang J Vezina G Undiagnosed Diseases Network, Ferreira CR DYRK1A Pathogenic variants in two patients with syndromic intellectual disability and a review of the literature. Mol Genet Genomic Med. (2020) 8(12):e1544. 10.1002/mgg3.1544 33159716 PMC7767569

[3] Ji J Lee H Argiropoulos B Dorrani N Mann J Martinez-Agosto JA DYRK1A haploinsufficiency causes a new recognizable syndrome with microcephaly, intellectual disability, speech impairment, and distinct facies. Eur J Hum Genet. (2015) 23(11):1473–81. 10.1038/ejhg.2015.71 25944381 PMC4613469

[4] Okazaki T Yamada H Matsuura K Kasagi N Miyake N Matsumoto N Clinical course of epilepsy and white matter abnormality linked to a novel DYRK1A variant. Hum Genome Variation. (2021) 8(1):26. 10.1038/s41439-021-00157-7 PMC8275604 34253714

[5] Ruaud L Mignot C Guët A Ohl C Nava C Héron D DYRK1A mutations in two unrelated patients. Eur J Med Genet. (2015) 58(3):168–74. 10.1016/j.ejmg.2014.12.014 25641759

[6] van Bon BWM Coe BP de Vries BBA Eichler EE. DYRK1A Syndrome. In: Adam MP Ardinger HH Pagon RA, et al., editors, Seattle: GeneReviews; University of Washington. (2015). Available at: https://www.ncbi.nlm.nih.gov/books/NBK333438/ (published online March 17). 26677511

[7] Méjécase C Way CM Owen N Moosajee M. Ocular phenotype associated with DYRK1A variants. Genes (Basel). (2021) 12(2):234. 10.3390/genes12020234 33562844 PMC7915179

[8] Earl RK Turner TN Mefford HC Hudac CM Gerdts J Eichler EE. Clinical phenotype of ASD-associated DYRK1A haploinsufficiency. Mol Autism. (2017) 8(1):54. 10.1186/s13229-017-0173-5 29034068 PMC5629761

[9] Qiao F Shao B Wang C Wang Y Zhou R Liu G A de novo mutation in DYRK1A causes syndromic intellectual disability: a Chinese case report. Front Genet. (2019) 10:1194. 10.3389/fgene.2019.01194 31803247 PMC6877748

[10] Bronicki LM Redin C Drunat S Piton A Lyons M Passemard S Ten new cases further delineate the syndromic intellectual disability phenotype caused by mutations in DYRK1A. Eur J Hum Genet. (2015) 23(11):1482–7. 10.1038/ejhg.2015.29 25920557 PMC4613470

[11] Schaeffer WJ Mahmood SMJ Vermillion SA Sweet R Haas NL. Splenic volvulus of a wandering spleen. Am J Emerg Med. (2021) 41:265.e1–3. 10.1016/j.ajem.2020.08.048 33041134

[12] Koliakos E Papazarkadas X Sleiman MJ Rotas I Christodoulou M. Wandering spleen volvulus: a case report and literature review of this diagnostic challenge. Am J Case Rep. (2020) 21:e925301. 10.12659/AJCR.925301 32868755 PMC7483514

[13] Wang Z Zhao Q Huang Y Mo Z Tian Z Yang F Wandering spleen with splenic torsion in a toddler: a case report and literature review. Medicine. (2020) 99(37):e22063. 10.1097/MD.0000000000022063 32925740 PMC7489642

[14] Reisner DC Burgan CM. Wandering spleen: an overview. Curr Probl Diagn Radiol. (2018) 47(1):68–70. 10.1067/j.cpradiol.2017.02.007 28385371

[15] Okui M Ide T Morita K Funakoshi E Fumiaki I Kiyokazu O High-level expression of the Mnb/Dyrk1A gene in brain and heart during rat early development. Genomics. (1999) 62(2):165–71. 10.1006/geno.1999.5998 10610708

[16] Rahmani Z Lopes C Rachidi M Delabar JM. Expression of the Mnb (dyrk) protein in adult and embryonic mouse tissues. Biochem Biophys Res Commun. (1998) 253(2):514–8. 10.1006/bbrc.1998.9803 9878567

[17] Rammohan M Harris E Bhansali RS Zhao E Li LS Crispino JD. The chromosome 21 kinase DYRK1A: emerging roles in cancer biology and potential as a therapeutic target. Oncogene. (2022) 41(14):2003–11. 10.1038/s41388-022-02245-6 35220406 PMC8977259

