# Corrigendum: Gut and spleen anomalies associated with DYRK1A syndrome

**DOI:** 10.3389/fped.2024.1372269

**Published:** 2024-02-16

**Authors:** I. Infantino, F. Tocchioni, M. Ghionzoli, R. Coletta, F. Morini, A. Morabito

**Affiliations:** ^1^ Department of Neurosciences, Psychology, Drug Research and Child Health (NEUROFARBA), University of Florence, Florence, Italy; ^2^ Department of Pediatric and Neonatal Surgery, Meyer Children’s Hospital IRCSS, Florence, Italy; ^3^ School of Health and Society, University of Salford, Salford, United Kingdom

**Keywords:** spleen abnormalities, gut abnormalities, DYRK1A, intestinal obstruction, splenectomy

A Corrigendum on Gut and spleen anomalies associated with DYRK1A syndrome Corrigendum on: Infantino I, Tocchioni F, Ghionzoli M, Coletta R, Morini F and Morabito A (2023) Case Report: Gut and spleen anomalies associated with DYRK1A syndrome. Front. Pediatr. 10:936732. doi: 10.3389/fped.2022.936732

**Incorrect Affiliation**

In the published article, there was an error in affiliation 2. Instead of “Meyer's Children Hospital, Department of Pediatric Surgery, University of Florence, Florence, Italy”, it should be “Department of Pediatric and Neonatal Surgery, Meyer Children's Hospital IRCSS, Florence, Italy”.

The authors apologize for this error and state that this does not change the scientific conclusions of the article in any way. The original article has been updated.

**Additional Affiliations**

In the published article, there was an error regarding the affiliations for R. Coletta. As well as having affiliation 1, they should also have Department of Pediatric and Neonatal Surgery, Meyer Children's Hospital IRCSS, Florence, Italy and School of Health and Society, University of Salford, Salford, UK.

There was an error regarding the affiliations for F. Morini. As well as having affiliation 1, they should also have Department of Pediatric and Neonatal Surgery, Meyer Children's Hospital IRCSS, Florence, Italy.

There was an error regarding the affiliations for A. Morabito. As well as having affiliation 1, they should have Department of Pediatric and Neonatal Surgery, Meyer Children's Hospital IRCSS, Florence, Italy and School of Health and Society, University of Salford, Salford, UK.

The authors apologize for this error and state that this does not change the scientific conclusions of the article in any way. The original article has been updated.

